# Facile Carbon Fixation to Performic Acids by Water-Sealed Dielectric Barrier Discharge

**DOI:** 10.1038/srep14737

**Published:** 2015-10-06

**Authors:** Mitsuo Kawasaki, Tatsuo Morita, Kunihide Tachibana

**Affiliations:** 1 Department of Molecular Engineering, Kyoto University, Katsura, Kyoto 615-8510, Japan; 2 PM Dimensions Corporation, 3-17-7 Goryo Minegado-cho, Kyoto 610-1103, Japan; 3 Osaka Electro-Communication University, Neyagawa, Osaka 572-8530, Japan

## Abstract

Carbon fixation refers to the conversion of carbon dioxide (CO_2_) to organic materials, as commonly performed in nature through photosynthesis by plants and other autotrophic organisms. The creation of artificial carbon fixation processes is one of the greatest challenges for chemistry to solve the critical environmental issue concerning the reduction of CO_2_ emissions. We have developed an electricity-driven facile CO_2_ fixation process that yields performic acid, HCO_2_OH, from CO_2_ and water at neutral pH by dielectric barrier discharge with an input electric power conversion efficiency of currently 0.2−0.4%. This method offers a promising future technology for artificial carbon fixation on its own, and may also be scaled up in combination with e.g., the post-combustion CO_2_ capture and storage technology.

There has been growing concern about the global warming due to the ever-increasing atmospheric CO_2_ concentration by the extensive burning of fossil fuels^1^. The current level of atmospheric CO_2_ concentration, as many scientists predict, potentially causes serious climate change to destroy many of the natural processes that human beings rely upon. Effective reduction of CO_2_ emissions is thus at the top of urgent international agenda in both political and scientific communities. To this end, it is one of the greatest challenges for chemistry to create artificial carbon fixation processes, of which the well-known examples are artificial photosynthesis by photocatalytic semiconductors^2^,^3^,^4^,^5^ and microbial CO_2_ fixation^5^,^6^,^7^. The success of these challenges rests not only upon the overall carbon fixation efficiency but also upon the robustness and commercial viability of the systems involved. It is most desirable that the process also leads to products with high commercial values. For example, the semiconductor artificial photosynthesis convers CO_2_ to CO, HCO_2_H, CH_4_, CH_3_OH, etc., preferably under so-called Z-scheme but in a highly complex manner^5^. It is questionable whether such systems can be scaled-up on a commercial basis and can keep the operation under intense sunlight for sufficiently long periods.

There is another field of active research closely related to the issue of carbon fixation; that is, either homogeneous^8^ or heterogeneous^9^, catalytic hydrogenation of CO_2_. CO_2_ is an abundant C_1_ resource to serve as a renewable feedstock for manufacturing a variety of useful compounds, but efficient catalysts are prerequisite to overcome the high thermodynamic and kinetic barriers associated with CO_2_. Among others, methanol has attracted widespread attention as a fuel derivable from CO_2_ by the catalytic hydrogenation according to,
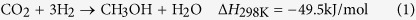
However, this is a type of fuel-to-fuel conversion representing a thermodynamically downhill reaction. This contrasts with the natural carbon fixation process with water as the hydrogen source. Besides, catalysts tend to be poisoned or deactivated by long time operation, and needs for recovery and regeneration may hamper commercial viability.

In this paper, we propose and demonstrate one promising, electricity-driven, facile CO_2_ fixation process that yields what we identify below as performic acid (PFA), HCO_2_OH, from CO_2_ and water by dielectric barrier discharge (DBD)^10^, which is elaborately designed to be operated in a water-sealed discharge gap with CO_2_ under atmospheric pressure; see Fig. 1 and Supplementary Fig. S1 online. The overall reaction, which is thermodynamically uphill, is given by,
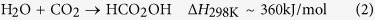
Here, we assumed that Δ*H* for HCOOH + (1/2) O_2_ → HCO_2_OH is approximately equal to that for H_2_O + (1/2) O_2_ → H_2_O_2_ in estimating the unknown enthalpy of formation of PFA.

PFA is known to have strong oxidizing properties, strongest among organic peracids. It has been used widely in organic synthesis^11^,^12^,^13^ and as a powerful sterilizer or disinfectant for wastewater treatment and in medical and food industries^14^,^15^,^16^,^17^. However, there have been no ways to prepare this material in pure state, and the conventional chemistry of PFA synthesis typically involves a sulfuric-acid-catalyzed equilibrium reaction in a highly concentrated mixture of FA and HP of the order of 10 M^14^.
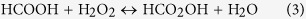


The high reactivity of the resultant strongly acidic solution connotes not only instability but also high handling dangers. Although the latter may be avoided by sufficient dilution, such diluted PFA solutions are still strongly acidic and a likewise acid-catalyzed reverse reaction (i.e., hydrolysis) predominates; see Supplementary Discussion and Fig. S2 online. By contrast, the relatively dilute aqueous peroxide solutions that are easily synthesized at neutral pH by the present method are unusually stable and yet exhibit much stronger oxidizing power than that of HP, which is difficult to account for by peroxides other than PFA or at least its close analogs. They not only remain intact for at least a month at room temperature, but also survive vaporization or condensation at ~70 °C or higher up to ~100 °C. Thus, much more concentrated solutions near 100 mM, corresponding to roughly ~1 wt%, can be obtained not only by prolonged DBD operation but also by post-DBD condensation.

## Results and Discussion

### Principle

**Figure 1:**
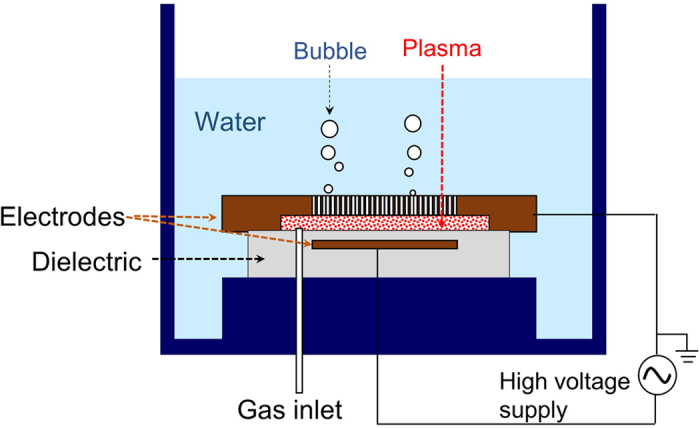
Schematic side view of water-sealed DBD apparatus. Not to scale. See Supplementary Fig. S1 for more details.

Figure 1 shows the specific configuration of DBD operated in water-sealed discharge gap with CO_2_ under atmospheric pressure. CO_2_ gas enters the narrow space of discharge gap at a constant flow rate and escapes into water through a number of small through-holes arranged in a honeycomb structure in the upper metal (Al) electrode. The constant CO_2_ flow and the resultant gas pressure effectively prevents water from leaking into the gap and hence stopping DBD, and also carries the CO_2_-fixation products away from the gap region into water. Water in this system also plays several crucial roles. Most importantly, the water/gas interface at each through-hole allows efficient encounters between the plasma electrons (e^−^*) with kinetic energy more than 5.1 eV and water probably in the vaporized form to yield H and OH radicals according to,
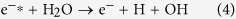
See Supplementary Discussion and Fig. S3 online for more details of this electron impact dissociation of water. Furthermore, the large volume of water serves as a reservoir for the CO_2_-fixation products and also as an effective coolant to remove the inevitable heat generated in the discharge gap. All these features are cooperatively-coupled for the facile formation of the product that we identify below as PFA from CO_2_ and water. We hereafter refer to this method as water-sealed DBD in CO_2_, WS-DBD in short.

The formation of PFA in the present system is believed to involve the following pathway for the reaction between the vibrationally excited CO_2_^v*^, readily produced in the DBD plasma, and the nascent H radical from reaction (4) to yield the formyl radical (see Supplementary Discussion and Fig. S4 online).
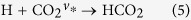
A barrierless attachment of OH radical to this intermediate complex then immediately results in production of PFA.
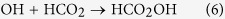
Other possible plasma reactions leading to other peroxide species would be:
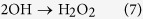

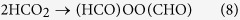

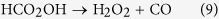
Reaction (9) represents a secondary disproportionation of PFA, which is more likely catalyzed by solid (metal or metal oxide) surfaces than to be a spontaneous (homogeneous) reaction.

The two-step radical reactions, (5) and (6), for production of PFA are quite simple and straightforward. Nevertheless, such a facile carbon fixation pathway has never been addressed before. This is probably because the formation of carboxyl radical, HOCO, is otherwise by far the dominant pathway in the reaction between CO_2_ and H^18^,^19^,^20^,^21^,^22^,^23^,^24^,^25^,^26^,^27^, where CO_2_ is rarely excited vibrationally and the activation energy is provided primarily by the kinetic energy of hot H atoms (see Supplementary Discussion and Fig. S4 online). The HOCO complex is the intermediate that then passes over to CO + OH, and provides no rational pathways to PFA. Note, in addition, that the collision between CO_2_ and the plasma electrons with excessively high energies (>12 eV) causes a dissociative excitation of CO_2_^28^, which cannot be linked to the formation of PFA either. Overall, WS-DBD in CO_2_ provides a unique reaction space where CO_2_ is highly excited vibrationally, particularly in the vending mode, and allowed at the same time to efficiently encounter the H radicals dissociated from water.

### Evidences for PFA as main product of carbon fixation

**Figure 2:**
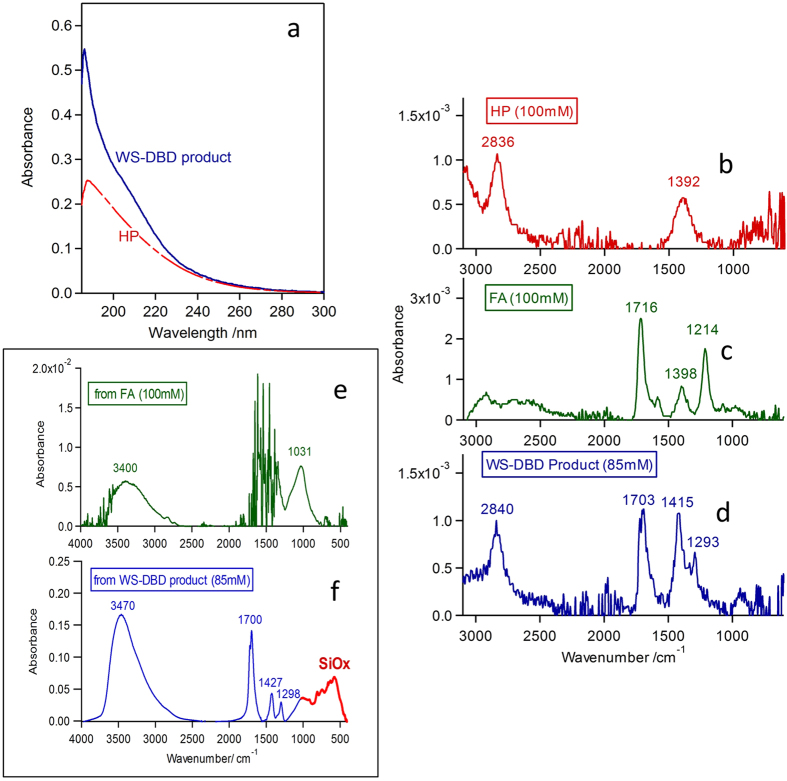
Spectroscopic evidences for carbon fixation to PFA by the present method. (**a**) Typical UV absorption spectrum of the WS-DBD product solution with total peroxide concentration of 0.95 mM. Dashed line in red represents, for reference, the spectrum taken for standard HP aqueous solution with the same total peroxide concentration. (**b–d**) ATR-FTIR spectra taken for HP (100 mM), FA (100 mM), and condensed WS-DBD product solution (85 mM). Reference was pure water. The numbers indicate the peak positions of major vibrational signals. (**e**,**f**) FTIR spectra taken for solid-like deposits on Si wafer from FA (100 mM) and WS-DBD product solution (85 mM). 0.125 mL of sample solution was drop-cast over the substrate area of 20 × 20 mm^2^ and dried at ~70 °C.

Figure 2 presents a collection of spectroscopic evidences for production of PFA as the main product of carbon fixation by the present method. Additional evidences that altogether support this conclusion are given in Supplementary Figs S5–S7 online and briefly addressed below. First of all, Fig. 2a shows a typical UV absorption spectrum of the WS-DBD product solution with the total peroxide concentration of 0.95 mM, as determined by the conventional iodometric titration method^29^. The dashed red line in Fig. 2a shows, for reference, the spectrum measured for an aqueous solution of pure HP with the same total peroxide concentration. The two spectra coincide well with each other in the long-wavelength region, dominated by the absorption due to the O–O bond common to HP and PFA. Whereas the WS-DBD product solution gives much stronger absorption in the short-wavelength region, due most likely to the *n*π^*^ transition of the carbonyl group coupled more-or-less strongly with the O–O group of PFA. Here we also refer to the UV absorption spectra of the standard PFA solution (Supplementary Fig. S2 online), which is an equilibrium mixture of PFA with HP and FA prepared according to the conventional chemical method^14^. We observe there a strong UV absorption characteristic of PFA, which is analogous to that in Fig. 2a, in the region of wavelength shorter than ~220 nm.

Next, the series of FTIR spectra shown in Fig. 2b–d, taken in the attenuated total reflection (ATR) mode for more concentrated solutions, give surer evidences for the formation of PFA by WS-DBD in CO_2_. As shown in Fig. 2b, HP exhibits two broad peaks at ~1390 cm^−1^ and ~2840 cm^−1^, in agreement with the literature^30^,^31^,^32^,^33^, particularly with the result of the analogous ATR-FTIR measurements of aqueous HP solutions by Voraberger and coworkers^32^, who assigned the ~1400 cm^−1^ band to O–H deformation and ~2800 cm^−1^ to O–H stretching vibration^34^, respectively. The three major peaks for FA in Fig. 2c also agree with the literature^35^,^36^, and are assigned to C=O stretching at 1716 cm^−1^, C–H bending at 1398 cm^−1^, and CO–COH deformation at 1214 cm^−1^, respectively.

The condensed WS-DBD product solution in Fig. 2d gives somehow intermediate features between HP and FA, in reasonable correlation with the molecular structure of PFA composed of only two functional groups; i.e., a formyl (HCO–) group on one end like FA and a hydroperoxy (–OOH) group on the other end like HP. The C=O stretching peak at 1703 cm^−1^, approximately half as strong as that for FA at 100 mM, thus represents the formyl group with its effective concentration comparable to the total peroxide concentration of 85 mM. The broad peak at 2840 cm^−1^, which resembles that of HP in Fig. 2c, comes from O–H stretching of the hydroperoxy group similar to that of HP. However, the vibrational features around 1400 cm^−1^ in Fig. 2d are quite different from the single broad peak for HP in Fig. 2b and do not match those of FA in Fig. 2c either. They thus must reflect O–H bending, C–H bending, and C–O–O deformation associated with the combination of formyl and hydroperoxy groups in PFA. The overall spectral features of Fig. 2d thus justify that the WS-DBD product is composed mainly of PFA. For reference, we have also taken ATR-FTIR spectra for the standard PFA solution (see Supplementary Fig. S5 online), but we could not observe any extra IR bands apart from those associated with the coexistent FA and HP. This also seems to be consistent with the fact that PFA gives intermediate vibrational features between FA and HP. The strong IR bands of FA mask them when it is coexistent in a large extra amount.

The high stability of the WS-DBD product solution also allowed a flow-injection-analysis time-of-flight Mass spectrometry (FIA-TOF MS) analysis (see Supplementary Fig. S6 online). The spectra taken in the electron-spray-ionization negative mode revealed a characteristic and strong peak at *m*/*z* = 62 irrespective of the fragmentor voltages. This peak is most probably due to the molecular ion of PFA, CHO_2_OH^−^. Furthermore, we made a detailed kinetic analysis of catalase-catalyzed decomposition of peroxides for a series of WS-DBD product solutions, to which an extra HP was deliberately added in varied molar ratios (see Supplementary Fig. S7 online). The coexisting HP undergoes a significantly faster selective decomposition by catalase. However, we clearly observed a resultant bi-exponential kinetics only for samples with an extra HP deliberately added afterwards. This means that the WS-DBD product solution itself had indeed a minor content of HP.

All of the above-noted evidences thus make us to be sure that the main product of the new carbon fixation pathway offered by the present method is PFA. However, co-presence of HP from reaction (6) and/or (9) or elsewhere, at a minor level of e.g.,~10% in molar ratio is still not totally ruled out on the present experimental grounds. As for diformyl peroxide from reaction (8), it should be a more minor product because DFP does not posses the O–H group on either side of the O–O bond to account for the 2840 cm^−1^ IR band discussed above.

The unique property of the WS-DBD product solution with PFA as the main ingredient is further corroborated in a little different perspective by the FTIR spectra presented in Fig. 2e,f. They have been taken in the transmission mode for solid-like deposits on Si wafer drop-cast from 100 mM FA for reference (Fig. 2e) and from 85 mM WS-DBD product solution (Fig. 2f), and dried at ~70 °C. Note that the 10-fold expanded vertical scale of Fig. 2e means that a major portion of FA drop-cast from the 100 mM solution volatilized in the drying step. Furthermore, the noisy signals around 1500 cm^−1^ in Fig. 2e strongly suggest that the residual FA on the Si wafer is adsorbed as formate. By contrast, in Fig. 2f, the characteristic features in the region from 1200 to 1800 cm^−1^ are very similar to those in Fig. 2d, indicating that PFA has made a solid deposit while retaining the almost same structure as that in solution. There is also no question that the strong broad peak around 3500 cm^−1^ in Fig. 2f is due to the O–H group, which strengthens that DFP is a minor constituent in the WS-DBD product solution.

### Strong oxidizing power of WS-DBD product

**Figure 3:**
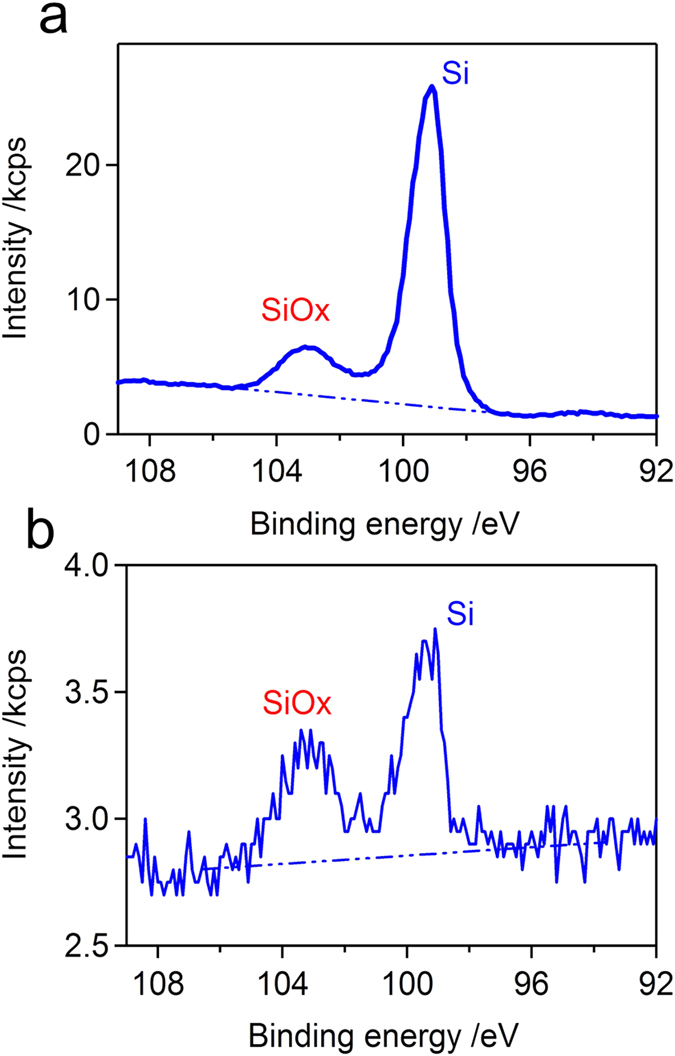
Oxidation of Si wafer surface by the WS-DBD product. (**a**) Si 2p core-level XPS spectrum taken for a bare Si water, showing a minor SiOx signal due to the native oxide as compared to Si signal from the bulk. (**b**) The solid deposit from the WS-DBD product solution on the Si wafer strongly attenuated the underlying SiOx and Si signals. A largely increased SiOx/Si intensity ratio indicates a substantial increase of the surface oxide thickness of the Si wafer.

Importantly, the relatively strong vibrational manifolds below 1000 cm^−1^ in Fig. 2f largely come from silicon oxides, representing substantial oxidation of the Si wafer by PFA. The oxidative change in the sub-surface chemical composition of Si wafer was also verified by X-ray photoelectron spectroscopy (XPS) analysis; see Fig. 3. XPS offers a sensitive tool to study the surface and/or sub-surface chemical composition of arbitrary solids^37^. In the case of Si substrate, the extent of surface oxidation is reflected upon the SiO_*x*_/Si intensity ratio in the Si 2p core-level spectra. Commercially available Si wafers, unless otherwise pre-etched chemically or by ion bombardment, possess a thin layer of native oxide, which gives rise to a relatively minor SiO_*x*_ signal as compared to the Si signal from the substrate bulk, as shown in Fig. 3a.

By contrast, a Si wafer with the solid deposits from the WS-DBD product solution gave much weaker Si 2p signals because the escape of the corresponding photoelectrons was strongly disturbed by the overlying solid deposit. However, this effect alone does not change the SiO_*x*_/Si intensity ratio, which is uniquely determined by the sub-surface chemical composition. The XPS spectrum taken in this condition, Fig. 3b, exhibited a remarkable increase in the SiO_*x*_/Si intensity ratio. This testifies to the impressively strong oxidizing power of the WS-DBD product solution as expected for PFA to substantially increase the thickness of the surface oxide layer. It should be also emphasized that, when a pure HP solution of 100 mM was drop-cast and dried on the Si wafer in the similar manner, essentially no IR signals were observed. This means that HP is not only more volatile than FA but also lacks the ability to oxidize the Si wafer surface at all.

**Figure 4:**
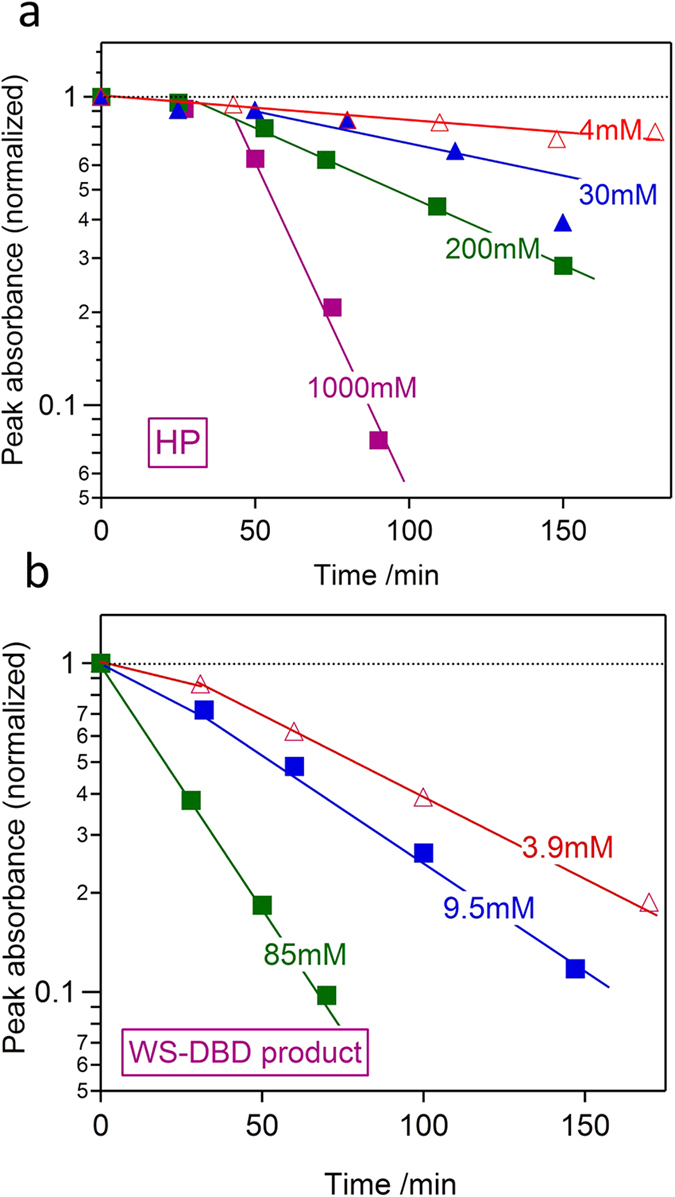
Kinetics of oxidative discoloration of methylene blue by HP and WS-DBD product. Normalized peak absorbance of initially 20 μM methylene blue aqueous solution is plotted as a function of time of oxidative discoloration at 70 °C for solutions containing (**a**) HP and (**b**) WS-DBD product at various concentrations. The WS-DBD product caused at least an order of magnitude faster oxidative discoloration of methylene blue.

Figure 4 further endorses the much stronger oxidizing capability of the WS-DBD product solution than that of HP, in terms of oxidative discoloration of a dilute (20 μM) methylene blue solution at ~70 °C. In the case of HP as the oxidant in Fig. 4a, there seemed to be some long induction period even at the highest HP concentration of 1000 mM. The discoloration reaction proceeded much more smoothly in the case of WS-DBD product as the alternative oxidant as shown in Fig. 4b, and overall we confirm at least an order of magnitude difference in the oxidizing capability between HP and WS-DBD product for this particular oxidation reaction under the given condition.

It should be emphasized again that the condensed WS-DBD product solution near 100 mM also keeps neutral pH and yet preserves such strong oxidizing power for at least a month or so. This strongly contrasts with the strongly-acidic conventional PFA solution mixed with FA and HP (see Supplementary Fig. S2 online), difficult to handle on a routine basis. There is no longer doubt that at least the major constituent in the WS-DBD product solution is PFA, as discussed above. The fact that such a strong and yet stable oxidant is easily manufactured at neutral pH adds further commercial value to the present CO_2_-fixation process.

### Efficiency of carbon fixation

Finally, the efficiency with which such useful oxidants are derived from the WS-DBD operation is worth considering. Specifically, the 0.95 mM product solution as examined in Fig. 2a was prepared at the AC line voltage and current of 70 V and 0.23 A, respectively, corresponding to power consumption of 0.95 kJ/min. The total volume of water was 500 mL and the total WS-DBD operation time was 2 h. The overall efficiency of PFA production per unit input energy was thus ~5 × 10^−6^ mol/kJ, which as compared to the enthalpy of formation of ~360 kJ/mol for PFA from CO_2_ and water, means the energy conversion efficiency of ~0.2%. This is still relatively small, but we trust that it will improve in the near future to the level of at least 1%. We already confirmed that an efficiency near 0.4% was readily achieved by simply warming up the water to temperatures above 50 °C, due most probably to the increase in the concentration of nascent H radicals produced by reaction (4) in the discharge gap. A substantial improvement is expected also with respect to the high frequency and high voltage power source to minimize the energy loss in the power line. The energy conversion efficiency of the level of ~1% also means that if we combine the present method with a solar panel with ~20% conversion efficiency, we will be able to use the solar energy for the given carbon fixation process at the overall solar energy conversion efficiency of ~0.2%. This favorably compares to that achieved in natural photosynthesis by plants and other autotrophic organisms.

As for throughput for the conversion of CO_2_ to PFA, the single small-scaled experimental apparatus affords conversion of 0.12–0.24 mL/min of CO_2_ under the low power supply of 0.95 kJ/min or ~16 W at the current energy conversion efficiency of 0.2–0.4%. As shown in Supplementary Fig. S1 online, the WS-DBD apparatus has a quite simple structure, and the combination of a dielectric-embedded working electrode with a corrosion-proof honeycombed metal plate ensures a sufficiently long operation time and robustness of the system. The fact that PFA forms at neutral pH also adds considerably to the durability of the reactor. Thus, the system will be scaled up easily both in the form of a single-operating unit with increased dimensions and in the form of multi-parallel operation of units lined up in a row or an array. The latter would be a more suitable form to combine the present method with e.g., the CO_2_ capture and storage technology^38^ for a mass production of PFA from waste CO_2_.

### Summary and Conclusion

The artificial carbon fixation technology has a large impact on the issue of global warming by the ever-increasing atmospheric CO_2_ concentration. We have developed a facile CO_2_-fixation pathway to performic acids based on the technique of water-sealed dielectric barrier discharge (WS-DBD). The effectively large plasma/water contact area provided by numerous through-holes in a honeycomb metal electrode enhances the decomposition of water by collision with the plasma electrons and the reaction of the resultant H radical with the vibrationally excited CO_2_ gives a hitherto unconceivable pathway to performic acid from CO_2_ and water. The reasonably high efficiency of carbon fixation by this method, together with other unique features, makes WS-DBD in CO_2_ a highly promising future technology for artificial carbon fixation.

## Methods

### General

The UV absorption spectra were measured with a UV-3600 spectrometer (Shimadzu Corp.) for samples filled in a 1 cm quartz cell. FTIR spectra were taken by using a Spectrum Two (Perkin Elmer) spectrometer either in transmission mode (on Si substrate) or in attenuated total reflection (ATR) mode along with a diamond universal ATR accessory. For XPS (X-ray photoelectron spectroscopy) analysis, ESCA-750 spectrometer (Shimadzu Corp.) was operated with Mg Kα radiation of 1254 eV. The plasma emission spectra were acquired by using an electronically-cooled multichannel analyzer (Hamamatsu Photonics, PMA-11, Model C5966-31) along with an input fiber optics having effective light-receiving area of 1 mm in diameter. A flow-injection-analysis (FIA) time-of-flight (TOF) Mass spectrometry (MS) of the WS-DBD product solution was done at Sumika Chemical Analysis Service, Ltd., by using an Agilent Technologies LC/MSD TOF system in the ESI (Electron-spray-ionization)-Negative mode with 1:1 water/methanol as the mobile phase.

### DBD Operation

The DBD plasma was generated in a narrow (0.5 mm) gap of sheet (30 × 30 mm^2^) between a dielectric-covered electrode and a metal electrode. The dielectric electrode was composed of borosilicate glass and iron nickel alloy buried therein. The dielectric barrier thickness was 1 mm. The metal electrode was made of aluminum, with a number of through-holes (0.75 mm in diameter) in a honeycomb arrangement. The whole electrode assembly was mounted at the bottom of a vessel (made of polyvinyl chloride resin) with total volume of approximately 3 L. (see Supplementary Fig. S1a). The electrode assembly fully sank under distilled water when the vessel was filled with water for more than ~300 mL. The feed gas (CO_2_) was supplied from outside of the vessel at a constant flowrate ranging from 20 to 500 mL/min. The 4–9 kV high voltage was supplied at the frequency of 20 kHz to the dielectric electrode from a resonated inverter transformer (Nagano Aichi Elec. AN-10).

## Additional Information

**How to cite this article**: Kawasaki, M. *et al.* Facile Carbon Fixation to Performic Acids by Water-Sealed Dielectric Barrier Discharge. *Sci. Rep.* **5**, 14737; doi: 10.1038/srep14737 (2015).

## Supplementary Material

Supplementary Information
