# Supplementary material for: Facile Carbon Fixation to Performic Acids by Water-Sealed Dielectric Barrier Discharge
Source: Sci Rep. 2015 Oct 6;5:14737. doi: 10.1038/srep14737 (PMC4593949; doi:10.1038/srep14737)
Supplement: Supplementary Information [file srep14737-s1.pdf]

Supplementary Information for

Facile Carbon Fixation to Performic Acids by Water-Sealed Dielectric Barrier Discharge

Mitsuo Kawasaki[1*†], Tatsuo Morita[2], Kunihide Tachibana[3]

[1]Department of Molecular Engineering, Kyoto University, Katsura, Kyoto 615-8510, Japan

[2]PM Dimensions Corporation, 3-17-7 Goryo Minegado-cho, Kyoto 610-1103, Japan

[3]Osaka Electro-Communication University, Neyagawa, Osaka 572-8530, Japan

*Correspondence to: mitsuo@sings.jp

† Current address: The Kyoto Technoscience Center, Rm.11, Yoshida-Kawaramachi, Kyoto 606-8305, Japan

Supplementary Materials and Methods

Chemicals. All chemicals were of special reagent grade and were used as received from Wako Pure Chemical Industries, Ltd., along with distilled water obtained from Osaka Yakken Co., Ltd.

Iodometric Titration. As one of the most popular redox titration methods,[1] the iodometric titration (or iodometry) allows easy quantitative determination of the total peroxide concentration in aqueous solutions where hydrogen peroxide (HP), performic acid (PFA), and other peracids are coexistent. Specifically, 0.15–1 mL of aqueous solution with unknown peroxide concentration was mixed with 1 mL of KI (1 M) and 0.1 mL of sulfuric acid (1 N) solution, and the resultant oxidation product, I_2, was titrated with 0.01 M aqueous solution of $Na_2S_2O_3$.

Catalytic Decomposition of Peroxides by Catalase.[2, 3] A total of 3 mL of aqueous peroxide solution (4–10 mM) to be analyzed was first acidified to pH~3.5 by adding 50 μL of 2% (0.35 M) acetic acid solution. Then 50 μL of 0.002 wt% aqueous solution of liver catalase was added to initiate the catalytic decomposition of the peroxide species at room temperature. The given acidic condition was chosen so that the decomposition kinetics was most clearly differentiated between HP and the WS-DBD product. The reaction was halted at an arbitrary length of reaction time by adding 0.1 mL of sulfuric acid (1 N), followed by the iodometric titration of the residual peroxide concentration.

DBD Apparatus. Figure S1 shows a side-view photo of the DBD apparatus with the whole electrode assembly mounted at the bottom of a reaction vessel. The upper metal electrode was made of aluminum with a number of through-holes (0.75 mm in diameter) in a honeycomb arrangement (see Fig. S1b). A seemingly homogeneous blue-light emission from the resultant discharge plasma confined in the narrow gap region is shown in Fig. S1c.

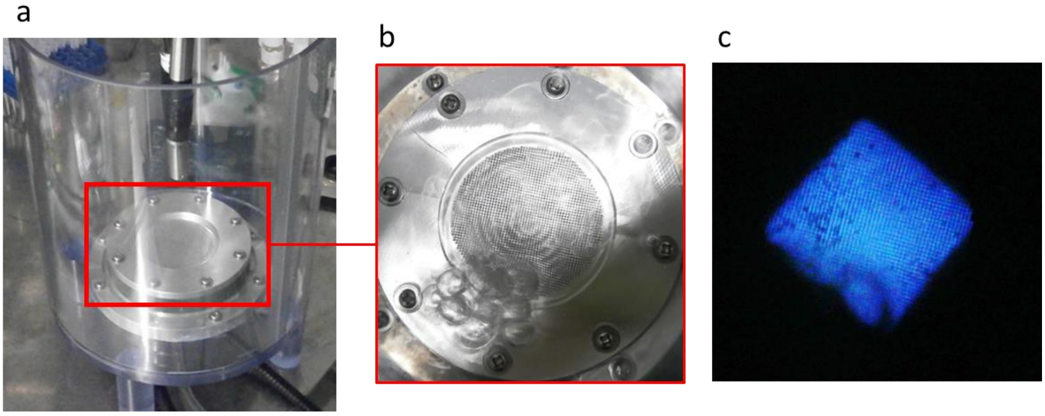

Supplementary Figure S1. Construction and operation of DBD apparatus. (**a**) A side-view of the reactor with the whole electrode assembly mounted at the bottom. (**b**) The top Al electrode with a large number of through-holes in a honeycomb arrangement. (**c**) A photo of blue-light emission from the discharge plasma, showing that the DBD discharge is confined in the gap region above the dielectric electrode (30×30 mm^2) and looks homogeneous to the eyes.

Supplementary Discussion

Stability assessment of standard PFA solutions. The PFA solution prepared following the standard protocol in the literature initially contained hydrogen peroxide (HP), formic acid (FA), and sulfuric acid (SA) for concentrations of 5.8 M, 9.9 M, and 0.77 M, respectively. The acid-catalyzed reaction[4] was allowed for approximately 1 h at 20°C, which converts roughly ~20% (~40%) of FA (HP) to PFA.[5] The resultant solution was quite unstable so that its UV absorption spectrum decayed monotonically by room-temperature aging as shown in Fig. S2a. The corresponding decrease in the total peroxide concentration is shown in Fig. S2b. By contrast, 500 to 1000-fold diluted PFA solutions exhibited a quite different behavior upon aging. Namely, the UV absorption spectrum exhibited clearly noticeable change by aging but only in the short-wavelength region as shown in Fig. S2c. Furthermore the total peroxide concentration remained constant in this case, indicating that the reverse reaction, i.e., a hydrolysis of PFA back to HP and FA, predominated in such diluted but still strongly acidic solutions cosisting of PFA, FA, HP, and sulfuric acid.

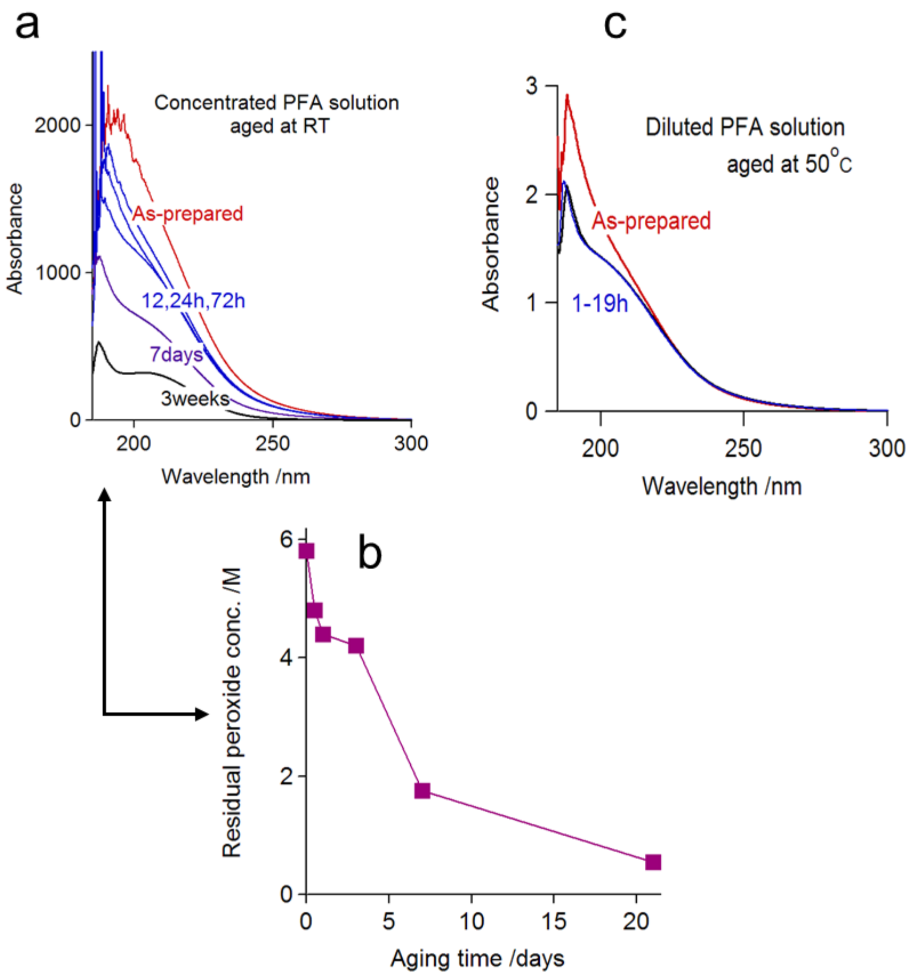

Supplementary Figure S2. Time courses of UV absorption spectra of concentrated and diluted PFA solutions prepared according to the standard chemical method.

(**a**) Irreversible change in UV spectrum taken for the concentrated (as-prepared) PFA solution as a function of aging time at room temperature. Each spectrum was taken for an aliquot of the solution, diluted by 500-fold immediately before the spectrum measurement, and the vertical axis of the resultant spectrum was re-scaled back to the real absorbance of the concentrated original solution. (**b**) The relationship between the residual total peroxide concentration and room-temperature aging time for the concentrated PFA solution. (**c**) Restricted change in UV spectra in the short-wavelength region for 1000-fold diluted PFA solutions as a function of aging time at 50℃. The total peroxide concentration did not change at all on the given timescale, indicating that the reverse reaction from PFA to HP and FA predominated in this case..

Plasma characterization for WS-DBD in CO_2. Figure S3 shows a typical emission spectrum from the WS-DBD plasma in CO_2 in our standard experimental condition. The emission from OH radicals at the wavelength (λ) of 309 nm ($A^2\Sigma - X^2\Pi$) is clearly visible in the figure, which is probably produced by the direct electron impact to H_2O, leading to the dissociative excitation; OH*+H with the threshold energy of about 9 eV.[6] The strongest emission series in the range from 300 to 400 nm are assigned to the $A^2\Pi - X^2\Pi$ bands of CO_2^+,[7] produced by the direct electron impact excitation with the threshold energy of about 18 eV,[8] while emission from the excited states of neutral CO_2 is absent. It is known that CO_2 is transparent for photons beyond λ~170 nm and the excited electronic states of CO_2 lie more than 8 eV above the ground state.[8, 9] Those electronic states are invariably dissociative with a high quantum yield of CO.[9] Thus no spectrum of neutral CO_2 is readily excited by the discharge in CO_2.[7] The emission from H radicals (H_α line at 656.3 nm) is also not noticeable in Fig. S3, of which the threshold energy for the dissociative excitation , OH+H*(n=3), of 18.5eV[6] is comparable to that of the CO_2^+ emission mentioned above, but the cross section is smaller by more than an order of magnitude at 20 eV.[6, 8] The other noticeable emission is assigned to the $B^1\Sigma - A^1\Pi$ bands of CO.[7] This emission probably arises from the electron impact to CO as the dissociation product of CO_2, which has much smaller threshold energy than the direct dissociative excitation of CO_2.

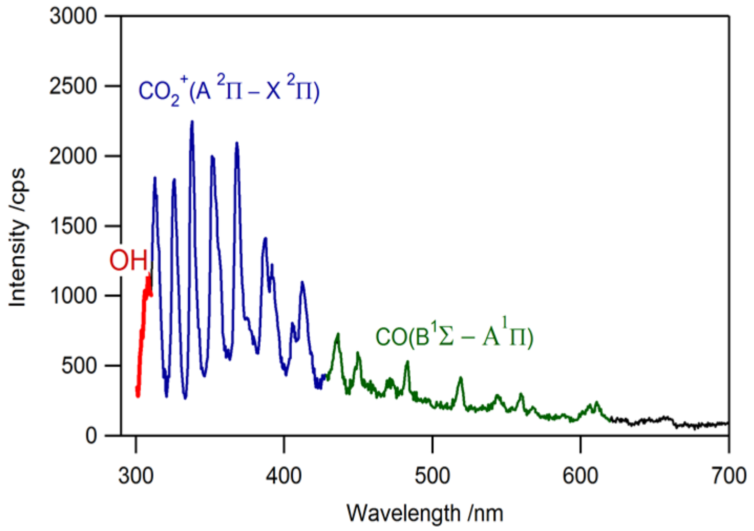

Supplementary Figure S3. Typical plasma emission spectrum acquired while the WS-DBD in CO_2 was under operation. The emission from OH radical (at 309 nm) is clearly visible, but not from H radical. There are no emissions from neutral CO_2, either. The strongest emission series arise from CO_2^+, along with minor emission series from CO.

Meanwhile, a typical DBD is known to be operated under the reduced electric field *E/N* (electric field strength divided by gas density) of 1×10^{-16} to 1×10^{-15} Vcm^2.[10, 11] In the present case, a crude estimate of *E/N* at the applied voltage of 7 kV across the 0.5 mm gas gap and the 1 mm glass dielectric becomes ~4×10^{-15} Vcm^2. Under this situation, the average electron energy estimated from the free software, Lxcat, becomes 0.1 to 9 eV.[12] Therefore, the ionization of CO_2 with the threshold energy of 13.8 eV[8] and the excitation of the CO_2^+ emission with the threshold energy of 18 eV[8] are taking place by the higher energy tail of the electron energy distribution function (EEDF). On the other hand, the dissociation of H_2O producing the ground-state OH and H radicals with the threshold energy of 5.1 eV[6] according to reaction (3) in the main text occurs with much higher probabilities by the main body of EEDF. It is also noted that the majority of lower energy electrons is imparted to the vibrational excitation of CO_2 especially at regions of lower *E/N* ranges.[8, 12, 13]

Energy diagram for the reaction of CO_2+H. Carboxyl radical (HOCO) and formyl radical (HCO_2) are two possible intermediates in the reaction between H and CO_2 . The corresponding simple and semi-quantitative energy-level diagram for these radicals relative to H+CO_2 is typically[14] as shown in Fig. S4, where only trans-isomer of HOCO is included. Although the pathway to HOCO has to surmount a considerably large (more than 100 kJ/mol) energy barrier, the reaction is exothermic and the attack of a hot H atom at either end O atom of CO_2 accounts for the exclusive preference of this pathway as established in the literature. However, the resultant HOCO complex is the intermediate that then passes over to CO + OH, and provides no rational pathways to PFA. The pathway to HCO_2 involves the attack of H atom on the central C atom and substantial change in molecular geometry from linear to C_{2v} symmetry. Thus this pathway would be allowed only for vibrationally excited CO_2 molecules. Once this radical formed, however, a small but considerable barrier with respect to the back-dissociation allows a barrierless attachment of OH radical to produce PFA.

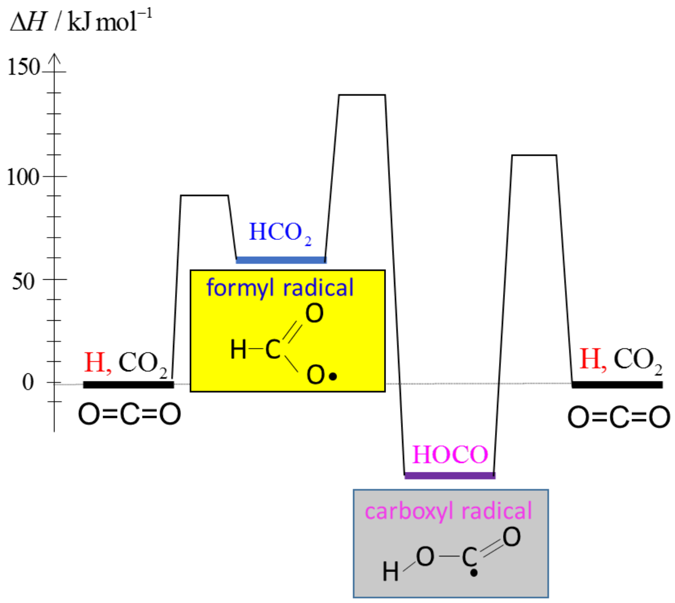

Supplementary Figure S4. Simplified semi-quantitative energy level diagram for the reaction between H and CO_2. Showing pathways to formyl and carboxyl radicals. The latter pathway has been shown to be exclusively dominant in most reaction conditions, but the formation of PFA in the present system is difficult to account for without the pathway to formyl radical as the intermediate.

ATR-FTIR spectroscopy of standard PFA solution. In addition to the UV spectroscopy measurements, as summarized in Fig S2, we also carried out an ATR-FTIR measurement for a standard PFA solution prepared according to the conventional chemical method. The measurement was done after 50 fold dilution with water for convenience. However, while the UV absorption spectra revealed a strong absorption due to PFA at the edge of far-UV wavelength region around ~200 nm, the FT-IR spectra, after subtraction of the sulfuric-acid peaks, were very similar to those derived for a simple mixture of HP and FA in the absence of sulfuric acid, as shown in Fig. S5. This fact suggests that PFA, which is composed of formyl (HCO-) and hydroperoxy (-O-OH) groups, affords intermediate IR features between FA and HP, which tend to be masked by the strong peaks due to FA, coexistent in a large extra amount in the standard PFA solution as examined here.

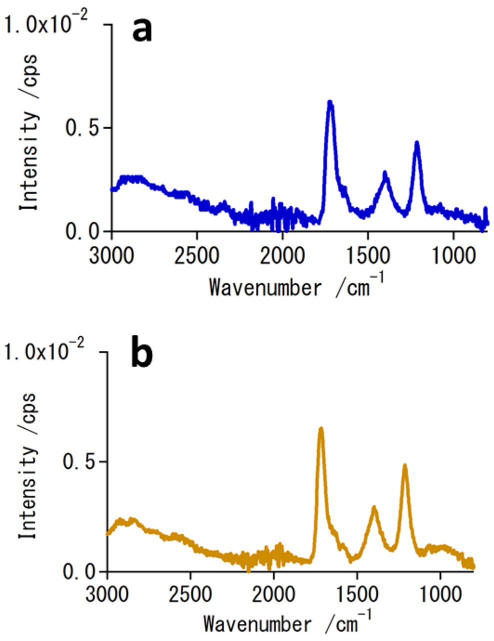

Supplementary Figure S5. FTIR analysis of standard PFA solution. (**a**) ATR-FTIR spectrum taken of a standard PFA solution diluted by 50 fold. The peaks originated from sulfuric acids were subtracted. (**b**) A comparative spectrum measured for a simple mixture of HP and FA in the equivalent concentrations but in the absence of sulfuric acid.

FIA-TOF MS analysis of WS-DBD product solution. In order to gain additional experimental support for the presence of PFA in the WS-DBD product solution, a sample with the total peroxide concentration of ~5 mM was subjected to FIA-TOFMS analysis in the ESI-Negative mode. The reference spectrum was taken for 0.17 mM standard carbonic acid solution. The observed MS patterns were strongly dependent on the fragmentor voltage and were not necessarily easy to make sure assignment of each peak. Nevertheless, the spectra shown in Fig. S6, taken at two different fragmentor voltages of 100 V and 180 V, clearly differentiated between the WS-DBD product solution and carbonic acid standard solution. Namely, in the former case (Figs. S6a and S6b) a strong peak at *m/z*=62, due probably to the molecular ion of PFA (CHO_2OH^-), appeared irrespective of the fragmentor voltage. The peak at *m/z*=61, strongest in Fig. S6a but missing in fig. S6b, may be due either to the deprotonated PFA (CHO_2O^-) or to the bicarbonate ion (HCO_3^-). By contrast, no peaks were visible at *m/z*=62 in the spectra of standard carbonic acid solution (Fig. S6c and Fig. S6d), for which the bicarbonate peak at *m/z*=61 was dominant at both fragmentor voltages.

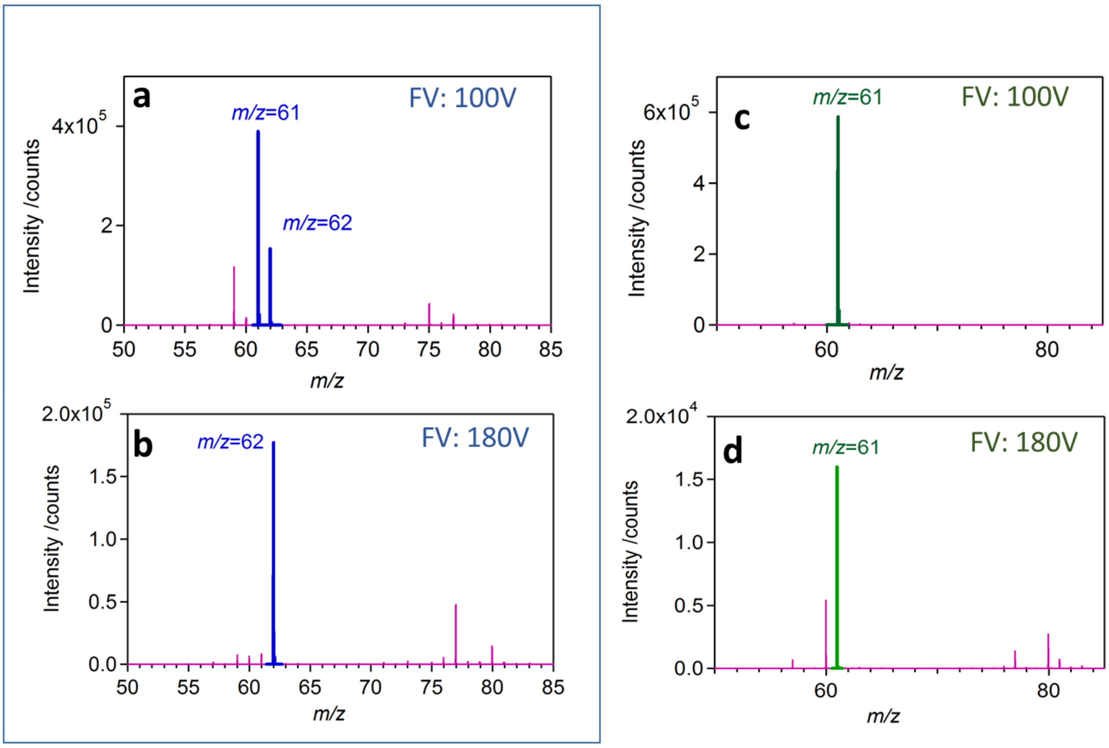

Figure S6. Negative-mode FIA-TOF MS spectra comparatively taken for WS-DBD product solution and standard carbonic acid solution. (a) The strongest and next strongest peaks are located at *m/z*=61 and *m/z*=62, which are probably due to the deprotonated PFA ion (HCO_2O^-) and the molecular ion of PFA (HCO_2OH^-), respectively. (**b**) There remains only the peak at *m/z*=62. (**c, d**) No peak is visible at *m/z*=62, and both at FV=100V and at FV=180V the strongest peak is located at *m/z*=61, undoubtedly due to the bicarbonate ion (HCO_3^-).

Kinetic analysis of peroxide decomposition by catalase. The WS-DBD product solution is believed to be composed mainly of PFA, but the relative content of potentially coexisting HP is not necessarily clear on the basis of the spectroscopic information alone. The selectively rapid decomposition of HP by catalase[2, 3] may help us to solve this problem. The reaction condition that we optimized for the present purpose was as specified above. First of all, Figure S7a shows how the decomposition rate in this condition differed between HP and WS-DBD product solutions, where the normalized residual peroxide concentration was plotted on semi-logarithmic scale as a function of reaction time. Here and below the initial total peroxide concentration was fixed to ~5 mM. The result indicates that the decomposition follows an approximately first-order reaction kinetics in both systems, but roughly with an order of magnitude larger rate constant for HP than that for the WS-DBD product. This also suggests that the amount of HP coexistent in the latter solution is indeed of negligible relative content. This conclusion is further supported by the kinetic behaviors observed for a series of WS-DBD product solutions intentionally mixed with HP in varied molar ratios; see Figs. S7b–S7d. It can be seen that the decomposition now follows a biexponential kinetics clearly uncovering a faster component representing the decomposition of HP mixed afterwards.

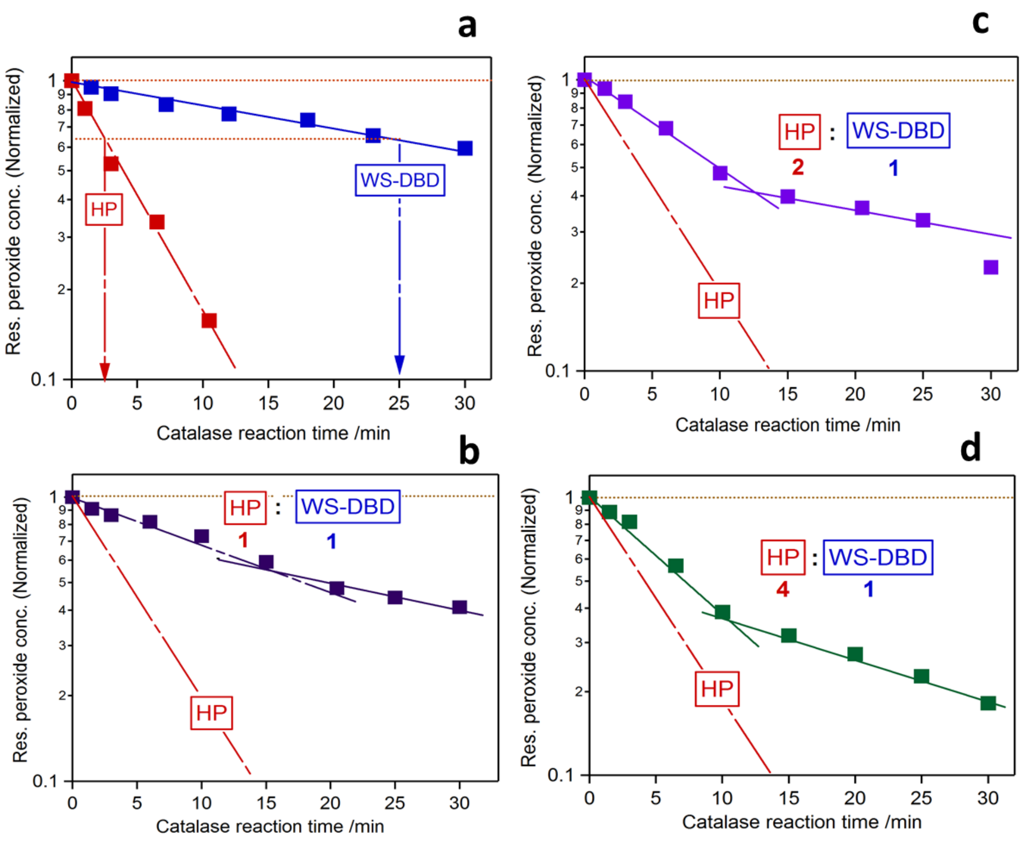

Supplementary Figure S7. Catalase-catalyzed decomposition kinetics measured for various samples consisting of WS-DBD product and intentionally added HP. (**a**) Comparison between WS-DBD product and pure HP solution (with total peroxide concentration of ~5 mM), demonstrating approximately single-exponential kinetics for both solutions. The rate constant for HP is an order of magnitude larger than that for the WS-DBD product. (**b**)–(**d**) The effect of addition of HP to WS-DBD product solutions in varied molar ratios, resulting in biexponential decomposition kinetics reflecting the coexistence of two different peroxide species.

References

S1. Harris, D. C. *Quantitative Chemical Analysis* (8th ed., W. H. Freeman & Co Ltd, 2010).

S2. Williams, J. The decomposition of hydrogen peroxide by liver catalase, *J. Gen. Physiol.* **11,** 309–337 (1928).

S3. Keilin, D. & Hartree, E. F. On the mechanism of the decomposition of hydrogen peroxide by Catalase, *P. Roy. Soc. Lond.* **124,** 397-405 (1938).

S4. Zhao, X., Zhang, T., Zhoub, Y. & Liu, D. Preparation of peracetic acid from hydrogen peroxide, part I: kinetics for peracetic acid synthesis and hydrolysis, *J. Mol. Catal. A-Chem.* **271,** 246–252 (2007).

S5. Gehr, R., Chen, D. & Moreau, M. Performic acid (PFA): tests on an advanced primary effluent show promising disinfection performance. *Water Sci. Technol.* **59,** 89–96 (2009).

S6. Itikawa, Y. & Mason, N. Cross section for electron collisions with water molecules. *J. Phys. Chem. Ref. Data* **34,** 1-22 (2005).

S7. Pearse, R. W. B. & Gaydon, A. G. *The Identification of Molecular Spectra* (4th ed., John Wiley & Sons, Inc., New York , 1976).

S8. Itikawa, Y. Cross sections for electron collisions with carbon dioxide. *J. Phys. Chem. Ref. Data* **31,** 749–767 (2002).

S9. Okabe, H. *Photochemistry of Small Molecules* (John Wiley & Sons, New York, 1978).

S10. Zhang, Y., Ying, J., Lu, N. & Shang, K. Diagnosis of electronic excitation temperature in surface dielectric barrier discharge plasmas at atmospheric pressure. *Plasma Sci. Technol.* **16,** 123–128 (2014).

S11. Mariana1, F. M. *et al*. NOx chemical reduction in a cylindrical DBD reactor: theoretical and experimental analysis. *Int. J. Plasma Environ. Sci. Technol.* **3,** 43–48 (2009).

S12. Hagelaar, G. J. M. & Pitchford, L.C. Solving the Boltzmann equation to obtain electron transport coefficients and rate coefficients for fluid models, *Plasma Sources Sci. Technol.* **14,** 722–733 (2005).

S13. Eliasson, B., Egli, W. & Kogelschatz, U. Modeling of dielectric barrier discharge chemistry. *Pure & Appl. Chem.* **66,** 1275–1286 (1994).

S14. E. H. Kim, S. E. Bradforth, D.W. Arnoid, R. B. Metz, D. M. Newmark, Study of HCO_2 and DCO_2 by negative ion photoelectron spectroscopy. *J. Chem. Phys.* **103,** 7801–7814 (1995).
